# Computational tools for copy number variation (CNV) detection using next-generation sequencing data: features and perspectives

**DOI:** 10.1186/1471-2105-14-S11-S1

**Published:** 2013-09-13

**Authors:** Min Zhao, Qingguo Wang, Quan Wang, Peilin Jia, Zhongming Zhao

**Affiliations:** 1 Department of Biomedical Informatics, Vanderbilt University School of Medicine, Nashville, TN 37232, USA; 2 Department of Cancer Biology, Vanderbilt University School of Medicine, Nashville, TN 37232, USA; 3 Department of Psychiatry, Vanderbilt University School of Medicine, Nashville, TN 37232, USA

## Abstract

Copy number variation (CNV) is a prevalent form of critical genetic variation that leads to an abnormal number of copies of large genomic regions in a cell. Microarray-based comparative genome hybridization (arrayCGH) or genotyping arrays have been standard technologies to detect large regions subject to copy number changes in genomes until most recently high-resolution sequence data can be analyzed by next-generation sequencing (NGS). During the last several years, NGS-based analysis has been widely applied to identify CNVs in both healthy and diseased individuals. Correspondingly, the strong demand for NGS-based CNV analyses has fuelled development of numerous computational methods and tools for CNV detection. In this article, we review the recent advances in computational methods pertaining to CNV detection using whole genome and whole exome sequencing data. Additionally, we discuss their strengths and weaknesses and suggest directions for future development.

## Background

Genomic variation is comprised of single nucleotide variants (SNVs), small insertions or deletions (indels), copy number variations (CNVs), and large structural variants (SVs); these variants range from single base changes to large chromosomal-level alterations [1]. CNV refers to a type of intermediate-scale SVs with copy number changes involving a DNA fragment that is typically greater than one kilobases (Kb) and less than five megabases (Mb) [2]. The importance of CNVs was first recognized by their prevalence in healthy individuals [3,4]. It is estimated that approximately 12% of the genome in human populations is subject to copy number change [5]. These pervasive CNVs in the genome are thought to have equal contributions to genetic variation in humans as another major type of variation, SNVs, which have long been considered the most abundant genetic variation in humans [6]. So far, approximately half of the reported CNVs overlap with protein-coding regions [3]. These gains and losses of gene copies might directly influence gene dosage within the CNV regions, which could result in a change of gene expression level. Similar to SNVs, CNVs do not necessarily have a negative effect on human health. However, among the large number of CNVs, some might have an association with, or directly involve in, diseases and phenotypes such as cancer and neuropsychiatric disorders [7,8]. Generally, CNVs include deletions, insertions, and duplications of genomic regions. Because the lengths of CNVs vary greatly, the current computational tools usually target a specific range of CNV sizes. The readers may be aware that this review focuses on all types of CNVs including CNV events less than 1 Kb, intermediate structural variants greater than 1 Kb, and large chromosomal events over 5 Mb.

The traditional approach to identify CNVs employs cytogenetic technologies, such as karyotyping and fluorescence *in situ *hybridization (FISH) [9]. In 2003, genome-wide detection of CNVs was achieved using more accurate array-based comparative genomic hybridization (arrayCGH) and single-nucleotide polymorphism (SNP) array approaches [10]; these approaches, however, have suffered from several inherent drawbacks, including hybridization noise, limited coverage for genome, low resolution, and difficulty in detecting novel and rare mutations [11,12].

Over the last few years, next-generation sequencing (NGS) has evolved into a popular strategy for genotyping and has included comprehensive characterization of CNVs by generating hundreds of millions of short reads in a single run [13]. Since 2005, several commercial platforms, including those from 454 Life Sciences, Illumina, Inc., and Life Technologies, have increased read generation throughput and base-calling accuracy unprecedentedly, enabling the sequencing of a whole human genome at a much lower cost and a faster turnaround time [12]. Compared to array-based approaches, where probes are predefined for limited genomic regions, short reads from NGS platforms are randomly sampled from the entire genome. The advantages of the NGS approach also include higher coverage and resolution, more accurate estimation of copy numbers, more precise detection of breakpoints, and higher capability to identify novel CNVs [1,14]. Taking these advantages into account, a diverse set of tools has been developed to detect CNVs based on different features that can be extracted from NGS data.

To provide guidelines for the rapidly growing number of CNV studies using NGS, this review presents a systematic investigation of the 37 currently available tools, which differ in computational strategies to pinpoint CNVs in whole genome sequencing (WGS) data. In addition, 11 *in silico *tools specific to whole exome sequencing (WES) data are discussed separately due to the challenges unique to CNV detection in WES data. Based on the discussion of the functions and limitations of current CNV-calling tools, and perspectives for future development, we mainly focus on: (i) the key features for CNV calling tools using NGS data, (ii) the key factors to consider before pipeline design, and (iii) developing combinatorial approach for more accurate CNV calling.

## Five strategies for CNV detection through NGS data

**Figure 1:**
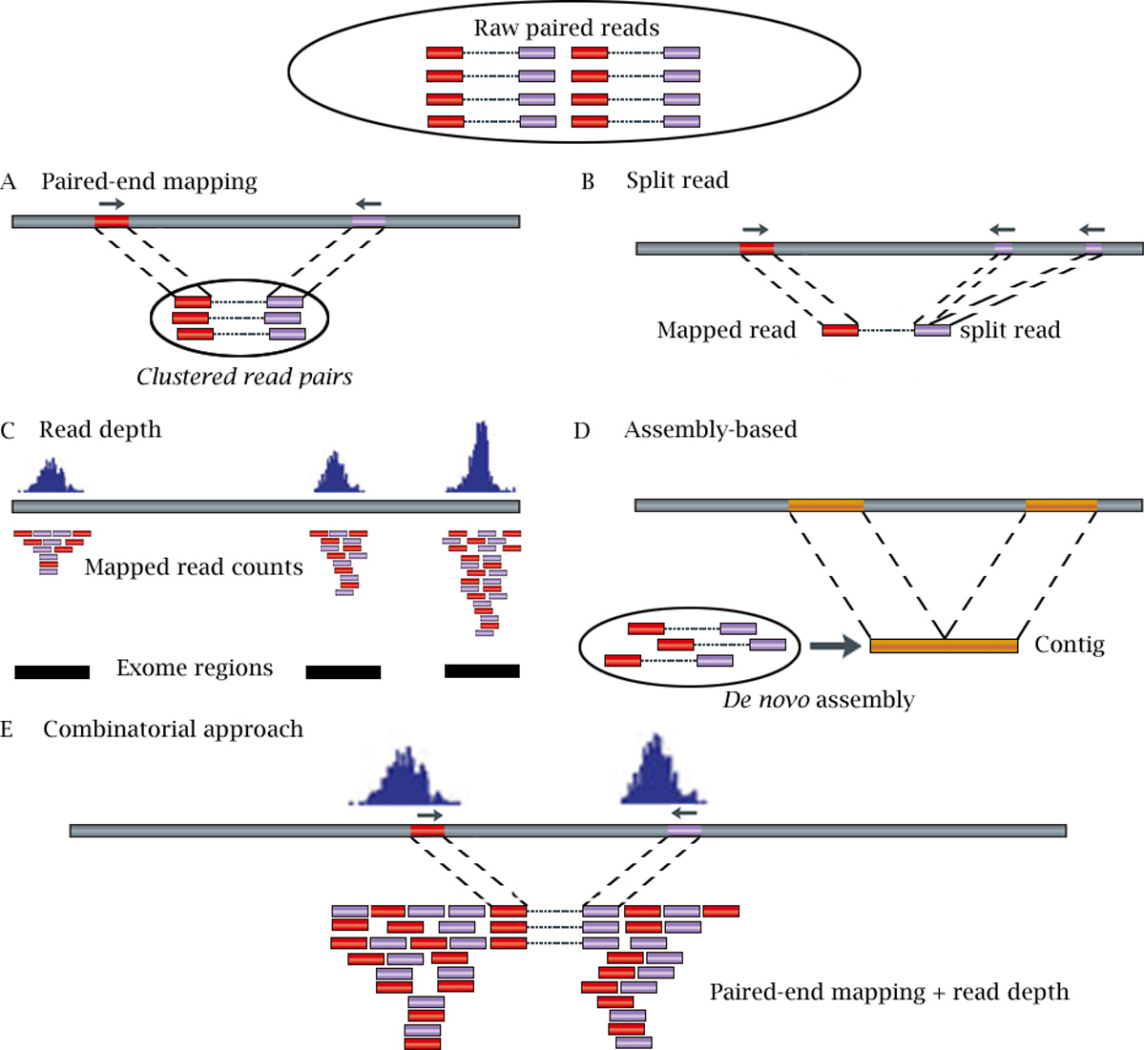
**Five approaches to detect CNVs from NGS short reads**. A. Paired-end mapping (PEM) strategy detects CNVs through discordantly mapped reads. A discordant mapping is produced if the distance between two ends of a read pair is significantly different from the average insert size. B. Split read (SR)-based methods use incompletely mapped read from each read pair to identify small CNVs. C. Read depth (RD)-based approach detects CNV by counting the number of reads mapped to each genomic region. In the figure, reads are mapped to three exome regions. D. Assembly (AS)-based approach detects CNVs by mapping contigs to the reference genome. E. Combinatorial approach combines RD and PEM information to detect CNVs.

Many SV-detecting methods can be applied to CNV identification. So far, the NGS based CNV detection methods can be categorized into five different strategies, including: (1) paired-end mapping (PEM), (2) split read (SR), (3) read depth (RD), (4) *de novo *assembly of a genome (AS), and (5) combination of the above approaches (CB) (Figure 1). Indeed, different strategies have their own advantages and limitations. Though there has been great progress in each category, none of the methods could comprehensively detect all types of CNVs. As summarized in Tables 1, 2, 3, 4, there are 6 PEM-based tools, 4 SR-based tools, 26 RD-based tools, 3 AS-based tools, and 9 tools for combinatorial approaches. Most PEM-, SR-, and CB-based tools are not specific to CNV detection but rather for SV identification, while the majority of RD- and AS-based tools are developed for the detection of CNVs instead of SVs. From the point of input materials, a majority (28) of the 48 tools listed in Tables 1, 2, 3, 4 accept SAM or BAM files as input. Specifically, most RD-based tools start from SAM/BAM files with depth information, while PEM- and SR-based tools can accept FASTQ files since they may not use read depth information. Among these tools, 32 were implemented using R and/or C/C++ computer languages, as they are convenient in incorporating statistical models, improving computational efficiency, and distributing across multiple operating systems. Note that most of these tools are standalone instead of online tools, except for visualization.

**Table 1:** Summary of paired-end mapping (PEM), split read (SR), and *de novo *assembly (AS)-based tools for CNV detection using NGS data

Method	URL	Language	Input	Comments	**Ref**.
PEM-based					
BreakDancer	http://breakdancer.sourceforge.net/	Perl, C++	Alignment files	Predicting insertions, deletions, inversions, inter- and intra-chromosomal translocations	[17]
PEMer	http://sv.gersteinlab.org/pemer/	Perl, Python	FASTA	Using simulation-based error models to call SVs	[18]
VariationHunter	http://compbio.cs.sfu.ca/strvar.htm	C	DIVET^a^	Detecting insertions, deletions and inversions	[20]
commonLAW	http://compbio.cs.sfu.ca/strvar.htm	C++	Alignment files	Aligning multiple samples simultaneously to gain accurate SVs using maximum parsimony model	[21]
GASV	http://code.google.com/p/gasv/	Java	BAM	A geometric approach for classification and comparison of structural variants	[65]
Spanner	N/A	N/A	N/A	Using PEM to detect tandem duplications	[59]
SR-based					
AGE	http://sv.gersteinlab.org/age	C++	FASTA	A dynamic-programming algorithm using optimal alignments with gap excision to detect breakpoints	[23]
Pindel	http://www.ebi.ac.uk/~kye/pindel/	C++	BAM /FASTQ	Using a pattern growth approach to identify breakpoints of various SVs	[22]
SLOPE	http://www-genepi.med.utah.edu/suppl/SLOPE	C++	SAM/FASTQ/MAQ^b^	Locating SVs from targeted sequencing data	[26]
SRiC	N/A	N/A	BLAT output	CalibratingSV calling using realistic error models	[25]
AS-based					
Magnolya	http://sourceforge.net/projects/magnolya/	Python	FASTA	Calling CNV from co-assembled genomes and estimating copy number with Poisson mixture model	[58]
Cortex assembler	http://cortexassembler.sourceforge.net/	C	FASTQ/FASTA	Using alignment of *de novo *assembled genome to build de Bruijn graph to detect SVs	[57]
TIGRA-SV	http://gmt.genome.wustl.edu/tigra-sv/	C	SV calls^c ^+ BAM	Local assembly of SVs using the iterative graph routing assembly (TIGRA) algorithm	N/A

^a^The specific input format for VariationHunter, including the reads with multiple alignments.

^b^File format from MAQ mapview.

^c^The file including the detected structure variations using other tools.

**Table 2:** Read depth (RD)-based tools for CNV detection using whole genome sequencing data

Tool	URL	Language	Input	Comments	**Ref**.
SegSeq^a^	http://www.broad.mit.edu/cancer/pub/solexa_copy_numbers/	Matlab	Aligned read positions	Detecting CNV breakpoints using massively parallel sequence data	[33]
CNV-seq^a^	http://tiger.dbs.nus.edu.sg/cnv-seq/	Perl, R	Aligned read positions	Identifying CNVs using the difference of observed copy number ratios	[31]
RDXplorer^b^	http://rdxplorer.sourceforge.net/	Python, Shell	BAM	Detecting CNVs through event-wise testing algorithm on normalized read depth of coverage	[28]
BIC-seq^a^	http://compbio.med.harvard.edu/Supplements/PNAS11.html	Perl, R	BAM	Using the Bayesian information criterion to detect CNVs based on uniquely mapped reads	[41]
CNAseg^a^	http://www.compbio.group.cam.ac.uk/software/cnaseg	R	BAM	Using flowcell-to-flowcell variability in cancer and control samples to reduce false positives	[44]
cn.MOPS^b^	http://www.bioinf.jku.at/software/cnmops/	R	BAM/read count matrices	Modelling of read depths across samples at each genomic position using mixture Poisson model	[46]
JointSLM^b^	http://nar.oxfordjournals.org/content/suppl/2011/02/16/gkr068.DC1/JointSLM_R_Package.zip	R	SAM/BAM	Population-based approach to detect common CNVs using read depth data	[45]
ReadDepth	http://code.google.com/p/readdepth/	R	BED files	Using breakpoints to increase the resolution of CNV detection from low-coverage reads	[38]
rSW-seq^a^	http://compbio.med.harvard.edu/Supplements/BMCBioinfo10-2.html	C	Aligned read positions	Identifying CNVs by comparing matched tumor and control sample	[34]
CNVnator	http://sv.gersteinlab.org/	C++	BAM	Using mean-shift approach and performing multiple-bandwidth partitioning and GC correction	[40]
CNVnorm^a^	http://www.precancer.leeds.ac.uk/cnanorm	R	Aligned read positions	Identifying contamination level with normal cells	[32]
CMDS^b^	https://dsgweb.wustl.edu/qunyuan/software/cmds	C, R	Aligned read positions	Discovering CNVs from multiple samples	[47]
mrCaNaVar	http://mrcanavar.sourceforge.net/	C	SAM	A tool to detect large segmental duplications and insertions	[35]
CNVeM	N/A	N/A	N/A	Predicting CNV breakpoints in base-pair resolution	[42]
cnvHMM	http://genome.wustl.edu/software/cnvhmm	C	Consensus sequence from SAMtools	Using HMM to detect CNV	N/A

^a^Tools require matched case-control sample as input.

^b^Tools use multiple samples as input.

**Table 3:** Summary of bioinformatics tools for CNV detection using exome sequencing data

Tool	URL	Language	Input	Comments	**Ref**.
Control-FREEC^a^	http://bioinfo-out.curie.fr/projects/freec/	C++	SAM/BAM/pileup/Eland, BED, SOAP, arachne, psi (BLAT) and Bowtie formats	Correcting copy number using matched case-control samples or GC contents	[53]
CoNIFER^b^	http://conifer.sf.net/	Python	BAM	Using singular value decomposition to normalize copy number and avoiding batch bias by integrating multiple samples	[54]
XHMM^b^	http://atgu.mgh.harvard.edu/xhmm/	C++	BAM	Uses principal component analysis to normalize copy number and HMM to detect CNVs	[55]
ExomeCNV^c^	http://cran.r-project.org/src/contrib/Archive/ExomeCNV/	R	BAM/pileup	Using read depth and B-allele frequencies from exome sequencing data to detect CNVs and LOHs	[49]
CONTRA^c^	http://contra-cnv.sourceforge.net/	Python	SAM/BAM	Comparing base-level log-ratios calculated from read depth between case and control samples	[77]
CONDEX	http://code.google.com/p/condr/	Java	Sorted BED files	Using HMM to identify CNVs	[78]
SeqGene	http://seqgene.sourceforge.net	Python, R	SAM/pileup	Calling variants, including CNVs, from exome sequencing data	[79]
PropSeq^c^	http://bioinformatics.nki.nl/ocs/	R, C	N/A	Using the read depth of the case sample as a linear function of that of control sample to detect CNVs	[52]
VarScan2^c^	http://genome.wustl.edu/software/varscan	Java	BAM/pileup	Using pairwise comparisons of the normalized read depth at each position to estimate CNV	[50]
ExoCNVTest^b^	http://www1.imperial.ac.uk/medicine/people/l.coin/	Java, R	BAM	Identifying and genotyping common CNVs associated with complex disease	[56]
ExomeDepth^b^	http://cran.r-project.org/web/packages/ExomeDepth/index.html	R	BAM	Using beta-binomial model to fit read depth of WES data	[30]

^a^Control-FREEC accepts either matched case-control samples or single sample as input.

^b^Tools use multiple samples as input.

^c^Tools require matched case-control samples as input.

**Table 4:** Combinatorial bioinformatics tools for CNV detection using NGS data

Method	URL	Language	Input	Combination^a^	**Ref**.
NovelSeq	http://compbio.cs.sfu.ca/strvar.htm	C	FASTA/SAM	PEM+AS	[66]
HYDRA	http://code.google.com/p/hydra-sv/	Python	Discordant paired-end mappings	PEM+AS	[67]
CNVer	http://compbio.cs.toronto.edu/CNVer/	Perl, C++	BAM/aligned positions	PEM+RD	[61]
GASVPro	http://code.google.com/p/gasv/	C++	BAM	PEM+RD	[63]
Genome STRiP	http://www.broadinstitute.org/software/genomestrip/genome-strip	Java, R	BAM	PEM+RD	[62]
SVDetect	http://svdetect.sourceforge.net/	Perl	SAM/BAM/ELAND	PEM+RD	[60]
inGAP-sv	http://ingap.sourceforge.net/	Java	SAM	PEM+RD	[64]
SVseq	http://www.engr.uconn.edu/~jiz08001/svseq.html	C	FASTQ/BAM	PEM+SR	[73]
Nord et al.	N/A	N/A	N/A	RD+SR	[74]

^a^RD: read depth-based approach; PEM: paired-end mapping approach; SR: split read approach; AS: *de novo *assembly approach.

### Paired-end mapping approach

The feasibility of using NGS data to detect SVs/CNVs was first achieved by PEM methods [15]. To date, six tools were developed specifically based on PEM (Table 1). In addition, at least 8 CB tools incorporate PEM algorithms to improve accuracy (Table 4). Notably, PEM is only applicable to paired-end reads but not single-end reads [15]. In paired-end sequencing, the DNA fragments from the same library preparation protocol have a specific distribution of the insert size. PEM identifies SVs/CNVs from discordantly mapped paired-reads whose distances are significantly different from the predetermined average insert size (Figure 1a). PEM methods can efficiently identify not only insertions and deletions but also mobile element insertions, inversions, and tandem duplications. However, PEM is not applicable to insertion events larger than the average insert size of the genome library [16]. Another limitation of PEM is its inability to detect CNVs in low-complexity regions with segmental duplication.

Two different strategies have been used in PEM-based tools to detect SVs/CNVs, namely the clustering approach and the model-based approach. The difference lies in that the clustering approach employs a predefined distance to identify discordant reads, while the model-based approach adopts a probability test to discover the unusual distance between read pairs in comparison to the distance distribution in genome. For the clustering approach, a cluster often includes at least two read pairs (Figure 1a), which is the minimal requirement to ensure the accuracy of the predication of breakpoints and the SV/CNV sizes [16]. The tool BreakDancer provides both clustering-based and model-based approaches to detect various SVs [17]: its module BreakDancerMax is clustering-based while the other module, BreakDancerMini, uses a model-based approach to detect smaller insertions and deletions ranging from 10- to 100-base pairs (bps). PEMer is another tool that uses a clustering-based strategy to detect SVs [18]. It implements various cluster sizes to guide read clustering and uses a customized cutoff to detect discordant read pairs. The advantage of PEMer is that the read clusters obtained from different platforms and different parameterizations can be merged together.

Both BreakDancer and PEMer are based on the hard cluster method. That is, each read can only be assigned to one cluster, and the reads that can be mapped to multiple genomic coordinates are discarded, even if they are mapped with high quality. Because of this limitation, it is not feasible to detect SVs/CNVs in repetitive genomic regions. To overcome this, VariationHunter [19] was developed by introducing a soft clustering strategy, which allows short reads in multiple clusters, aiming to improve sensitivity. It proposes two algorithms to implement soft clustering of the discordant reads: VariationHunter-SC (Set Cover) and VariationHunter-Pr (Probabilistic). Using maximum parsimony, VariationHunter-SC can minimize the total number of genomic clusters by mapping each paired-end read to a particular SV. VariationHunter-Pr was developed to solve the problems present when the same paired end reads are mapped to multiple locations with equal support. Additionally, VariationHunter was extended to detect transposon insertion events from NGS data recently [20].

Aside from the single sample-based PEM algorithm, a multiple samples-based PEM algorithm was also implemented in commonLAW [21], a software that compares all the samples to a reference genome simultaneously to obtain more accurate SVs. Similar to VariationHunter-SC, commonLAW implements a maximum parsimony approach to detect SVs/CNVs. In other words, commonLAW works like a multiple alignment tool if we consider VariationHunter as a pairwise alignment method in this analogy.

### Split read-based approach

SR methods start from read pairs in which one read from each pair is aligned to the reference genome uniquely while the other one fails to map or only partially maps to the genome (Figure 1b). Those unmapped or partially mapped reads potentially provide accurate breaking points at the single base pair level for SVs/CNVs. SR methods split the incompletely mapped reads into multiple fragments. The first and last fragments of each split read are then aligned to the reference genome independently. This remapping step therefore provides the precise start and end positions of the insertion/deletion events. However, the SR-based approach heavily relies on the length of reads and is only applicable to the unique regions in the reference genome.

Pindel is the first SR-based method to identify breakpoints of large deletions (1 bp - 10 Kb) and medium-sized insertions (1 - 20 bps) [22]. Starting with unmapped or partially mapped reads from read pairs, this tool utilizes a string match approach to search unique substrings in minimum (the 5' end of the input reads) and maximum locations (the 3' end of the input reads) in which both could be completely mapped to the genome. Based on the minimum and maximum location, the reads are split into either two (deletion) or three (short intra-read insertion fragment in the middle) segments. Another SR-based tool, Alignment with Gap Excision (AGE), identifies SV breakpoints with base pair accuracy using more strict alignments with gap excision [23]. Using a local alignment algorithm similar to Smith-Waterman [24], AGE can simultaneously align two sequences accurately. Since this tool requires a predefined SV/CNV region as input to guide local alignment, it provides a complementary strategy to other tools in identifying SVs/CNVs. Similarly, the SR-based tool split-read identification, calibrated (SRiC), adopts gapped alignment to detect SVs [25]. Lastly, another SR-based tool, SLOPE, was developed for targeted sequencing data of a limited number of genomic regions of interest [26] (Table 1).

### Read depth-based approach

Mainly due to the accumulation of high-coverage NGS data, RD-based methods have recently become a major approach to estimate copy numbers. The underlying hypothesis of RD-based methods is that the depth of coverage in a genomic region is correlated with the copy number of the region, e.g., a gain of copy number should have a higher intensity than expected [27]. Compared to PEM and SR-based tools, RD-based methods can detect the exact copy numbers, which the former approaches are lacking because PEM/SR methods only use the position information. Moreover, RD-based methods can detect large insertions and CNVs in complex genomic region classes, which are difficult to detect using PEM and SR methods [28].

Currently, WGS and WES are the two major NGS platforms for DNA-sequencing (DNA-seq). WGS can determine the full spectrum of variants in the whole genome. WES is a more effective, targeted sequencing of protein coding regions in the genome, often with high coverage. In this section, we review the RD-based tools for WGS and WES separately, as they utilize different technologies and produce different data. However, both platforms generate similar raw data in the format of short reads and involve the mapping of short reads to the reference genome. The mapping results are then used to calculate read depth in both WGS and WES.

Generally, RD-based tools can be classified into three categories depending on the study design: single samples, paired case/control samples, and a large population of samples. When there is only a single sample, it is not possible to borrow information from other samples or the matched control. Therefore, a widely applied strategy in such cases is to estimate the read depth distribution using mathematical models and to discover regions with abnormal depth departing from the overall distribution. The copy numbers reported in such cases are more likely to be absolute numbers of copies. When there are pairs of matched samples, CNV detection can be leveraged on the matched control, which could serve as a "reference" genome. The copy numbers in such cases are reported more like relative copies compared to the reference control, instead of absolute copies. Finally, to detect CNVs from a population of samples, tools are developed using the overall mean of the read depth from multiple samples to discover the discordant copy numbers in each sample.

Basically, RD-based methods follow a four-step procedure to discover CNVs: mapping, normalization, estimation of copy number, and segmentation (Figure 1c). In the mapping step, short reads are aligned to the reference genome, and the read depth is calculated according to the number of mapped reads in predefined windows. The second step focuses on normalization and correction of potential biases in read depths mainly caused by GC contents and repeat genomic regions, among others. With the normalized read depths, the third step is to estimate the accurate copy number along the chromosome to determine the gain or loss. Lastly, the genomic regions with a similar copy number are merged to detect discordant copy number regions [29]. Notably, after mapping and normalization, the read depth data from NGS experiments are similar to the probe log ratios from arrayCGH data in the mathematical view. Therefore, some classical algorithms to detect CNV regions from arrayCGH data can be reused and modified to work on NGS read depth data.

#### Tools for whole genome sequencing data

Generally, RD-based tools define non-overlapping genomic windows, calculate read depths for these windows, and estimate copy numbers for each of them. Deeper read depth can not only increase the statistical power of CNV detection, but also help to infer the best size of a sliding window [30]. A moving window with a fixed width is the simplest way to divide a chromosome [31]. However, some genomic regions with a small read count may create a non-uniform fluctuant, which may result in high false positive detection. In CNAnorm, the authors suggested choosing the best size for sliding windows so that the number of reads mapped to each window is between 30 and 180 for WGS data [32]. Based on a user-defined false discovery rate (FDR), ReadDepth and mrCaNaVar can automatically set an appropriate size for a sliding window according to the mean number of reads in each window. RDXplorer proposed a novel algorithm using significance testing called EWT (Event-Wise Testing), which provides a high resolution for CNV detection using 100-bp windows [28]. A Z-score is calculated based on the number of reads mapped in each 100-bp window, according to a two-tailed normal distribution. Using a probabilistic approach, EWT was implemented to search unusual depth intervals with deletion and insertion events using corrected Z-scores. The tool SegSeq requires a predefined amount of reads mapped in a genomic window [33]. Similar to SegSeq, recursive Smith-Waterman-seq (rSW-seq) was developed for CNV detection in matched tumor-control samples [34]. Different from SegSeq, rSW-seq does not rely on predefined genomic windows. It first sorts reads according their genomics regions. Next, positive and negative weight values are assigned to tumor and control samples, respectively. The cumulative changes of assigned weight values are used to determine breakpoints that indicate copy number gains or losses.

Another key challenge for RD-based CNV detection is an efficient algorithm to segment the detected CNV regions after choosing the appropriate sliding window. Earlier methods such as mrCaNaVar and CNV-seq are implemented in a model-free style [31,35,36]. After obtaining the read depth, mrCaNaVar directly localizes segments with significantly different read depths to the overall read depth mean. The segments with three times the standard deviation over the mean of read depth are determined as duplication events. Similar criteria are applied to deletions. However, this model-free approach tends to have a high FDR even after correcting for GC-content and mapping biases.

Most of the methods use statistical models, such as Circular Binary Segmentation (CBS), Mean Shift-Based (MSB), Shifting Level Model (SLM), Expectation Maximization (EM), and Hidden Markov Model (HMM) for CNV detection (Table 2). These models assume that there is a sequence of segments (gain or loss) along each chromosome that could be detected using probability distribution functions with statistical measurements, such as the mean and variances of read depths. Compared with mode-free approach, approaches using mathematical models to detect CNVs generate more reliable results after filtering false positive regions.

The CBS method was originally developed for arrayCGH data to convert the noisy intensities into segments with equal copy numbers. Essentially, the algorithm recursively localizes the breakpoints by changing genomic positions until the chromosomes are divided into segments with equal copy numbers that are significantly different from the copy numbers from their adjacent genomic regions [37]. The first tool to introduce CBS in NGS data is SegSeq [33], which uses a case (e.g., cancer) versus control dataset to detect CNVs. At first, the copy number ratio of the two matched samples is calculated for each genomic window. Using the CBS algorithm, SegSeq identifies the boundaries of DNA fragments with changed copy numbers. ReadDepth is another RD-based CNV calling tool that uses the CBS approach to segment chromosomes [38]. However, it does not require paired samples; rather, it can be applied to a single sample. Notably, ReadDepth is able to discover epigenetic changes due to its capacity to process both regular and bisulfite-treated reads [38].

MSB [39] is another widely applied algorithm that was originally developed for CNV segmentation in arrayCGH data. In the MSB approach, the adjacent genomic windows with similar read depths are merged together along chromosomes. The breakpoints are reported when read depths of a sliding window are significantly discordant with the depths of the merged windows. Two software tools for NGS data, CNVnator [40] and BIC-seq [41], implement the MSB approach to choose the window size for read depth signal partitioning. CNVnator works on single individual samples to implement MSB. The key feature of CNVnator is its capability to detect CNVs in various sizes ranging from a few hundred bases to megabases. In contrast, BIC-seq, which also implements the MSB approach, works on matched pair samples instead of single samples [41].

CNVeM is a recently developed tool to determine CNVs from individual samples with all possible mapping information [42]. It utilizes mrsFAST to map reads, allowing up to 2 mismatches [43]. Those ambiguously mapped reads within each 300-bp window are used to estimate copy numbers using an EM algorithm. Instead of searching CNVs in a genomic region, the copy number of each base is calculated based on its mapped reads. Therefore, this algorithm can predict breakpoints in base pair resolution. Due to its ambiguous mapping strategy, CNVeM is also useful to distinguish the gain or loss for human paralog regions.

CNAseg is a representative tool utilizing HMM in the segmentation step to identify contiguous genomic regions with similar read depths [44]. The key feature of CNAseg is its powerful framework to control the false positive rate using read depth variability between different flow cells from case and control samples. JointSLM is another tool clustering the small abnormal genomic regions using an HMM approach [45]. However, JointSLM does not rely on single or paired samples but builds a statistical model based on multiple samples. Taking advantage of the simultaneous analyses of multiple samples, JointSLM works better than the EWT algorithm implemented in RDXplorer [28] in terms of detecting small CNV regions as short as 500 bp. In addition, higher sensitivity can be achieved by JointSLM with more samples [45].

JointSLM is not the only tool to utilize multiple samples to detect CNVs. Two additional tools, cn.MOPS and correlation matrix diagonal segmentation (CMDS), are proposed to increase statistical power and decrease computational burden based on a multiple samples approach. cn.MOPS proposed a data processing pipeline using a mixture of Poisson models to reduce the FDR in CNV detection [46]. By modelling read depths across multiple samples in each genomic region, this tool can remove the effect of read depth variation along chromosomes. Another multiple sample-based tool, CMDS, was developed to identify CNVs using a between-chromosomal-site correlation analysis [47]. CMDS initially takes all copy numbers from the same genomic regions from multiple samples as input. The correlation coefficients between two different genomic regions across multiple samples are calculated. The diagonal transformation and statistical tests are applied on the correlation coefficients to identify focal events (CNVs). Though CNVnator also claims its ability to discover CNVs from families and populations, it calls CNVs in each sample before the cross-sample comparison [40]. Compared with other tools, multiple sample-based tools often gain high sensitivity and a lower false positive rate [46].

The different RD-based tools have their own limitations and advantages. In a recent comparison of different RD-based tools [29], EWT-based methods (e.g., RDxplorer) and SLM-based methods (e.g., JointSLM) showed the best performance with both high sensitivity and specificity. For large windows over 50 bp, the CBS-based tool (ReadDepth) and MSB-based tool (CNVnator) performed well, but their accuracy was decreased when dealing with smaller windows less than 10 bp. The HMM based tool, CNASeg, could obtain better CNV calling on high coverage data but performs worse on low coverage data. In terms of the size of detected CNVs, RDxplorer could detect CNVs with a length of ~500 bp. However, ReadDepth and CNVnator can identify larger events ranging between 2-5 Kb.

#### Tools for CNV detection through WES data

Due to investigators' interest in coding gene regions and its lower cost, WES is widely applied to Mendelian as well as other complex diseases [48]. WES has the potential to rapidly detect single nucleotide mutations and CNVs in human coding regions. The inherent limitation of WES is that it produced reads only covering 1-2% of the human genome based on the currently available exome extraction kits. The full spectrum of CNVs and breakpoints may not be completely characterized. In addition, many large CNVs and cross-chromosome events may not be detected. Nevertheless, CNV detection based on WES data may give a quick insight into CNV patterns for a specific disease or phenotype. The analysis tools reviewed below may help establish rational pipelines to identify various CNVs in matched case-control pairs, multiple disease samples, and common CNVs in large populations.

In contrast to WGS, WES data have higher depth for targeted regions, which is ideal for more accurate CNVs using an RD-based calling approach. However, due to differing capture efficiency, the depth from different genomic regions may vary substantially and should be considered in the downstream analysis of CNV calling. First, due to inconsistent capture efficiency, there might be regions that are poorly sequenced, which requires pre-processing for WES data. Second, the assumption of normal distribution may no longer be valid due to the biases regarding read depth distribution. Third, due to the discontinuation of genomic regions, most CNV breakpoints could not be detected. Finally, the widely applied segmentation algorithms to merge windows in WGS may not be applicable in WES data due to the non-continuous distributions of the reads.

To keep pace with the flood of WES data, at least 11 RD-based CNV calling tools have been developed to address the unique challenges of WES data (Table 3). Similar to the methods for WGS data, the methods for WES data may also rely on matched case and control samples or single samples to detect discordant CNV profiles [49]. ExomeCNV focuses on regions whose coverage is higher than a predefined minimum depth and assumes Gaussian distribution to characterize the read depth from WES. Similarly, VarScan 2 requires a minimum coverage of 3× for each captured region from WES data to reduce potential false positives [50]. Based on the relative CNVs between the case and control samples, VarScan 2 adopts a CBS algorithm implemented by DNAcopy [51] for segmentation. Unlike directly modelling the log-ratios of the coverage between case and control samples for each region, the tool PropSeq implements a regression model to represent the read depth of a given region in the case sample as a function of its counterpart in the control sample [52].

Recently, in the work of ExomeDepth, the authors argued that the Gaussian assumptions may not completely characterize the read count from WES data due to the technical noise from library preparation, capture, and sequencing. Rather, ExomeDepth shows that the beta-binomial model could fit WES data better than the Gaussian model. In addition, because the ExomeDepth does not rely on the CNVs in the reference sample, it is useful in the detection of rare CNVs [30]. Another flexible tool, Control-FREEC, is able to call CNVs from WGS and WES data with or without control samples [53]. An additional convenience of Control-FREEC is its acceptance of almost all available formats currently used in NGS data rather than being limited to SAM/BAM files.

All the above CNV detection methods are designed to detect CNVs from NGS data for small-scale samples, paired or not, and are especially prevalent for cancer and normal pairs. Another scenario in WES data is separate, large-scale sequencing of a disease population and a control population. Similar to the trends in CNV calling for WGS data, more and more tools utilize multiple samples with WES data to increase sensitivity and reduce the FDR. These tools, such as CoNIFER (copy number inference from exome reads), XHMM (exome hidden Markov model), and ExoCNVTest, fit particularly well for high-resolution and large population-based studies to detect rare and common CNVs. CoNIFER was the first developed to deal with rare CNVs from multiple samples [54]. It starts by calculating the average signal intensity for each captured region in each of the samples. Using the singular value decomposition (SVD) approach, CoNIFER normalized absolute copy numbers from each sample to discover rare CNVs. At almost the same time as CoNIFER was released, another group reported the tool XHMM, which adopts a similar concept to normalize the raw read depth data but uses principal-component analysis (PCA) [55]. Following the recalibration of read depth, XHMM implements an HMM model to find the significant discordant CNVs. Both tools utilize a large sample size to detect CNVs while reducing potential technical noises from exome capture reactions and batch effects by removing the major variances detected in SVD or PCA. The main advantages of these two methods include that they are control-free and can be applied to large disease cohorts. To date, ExoCNVTest is the only tool to specifically detect common CNVs from WES data [56]. After normalizing the read depth, ExoCNVTest calculates the local first principal component (FPC) on the read depth for each sample. Then, the local FPCs are used to discover the global principal components, which may record the CNV information. The association between global principal components and particular genomic regions is carried out to detect the absolute copy number genotypes with case-control samples.

### Assembly-based approach

Different from the RD, PEM and SR approaches that first align NGS reads to a known reference genome before the detection of CNVs, the AS-based methods first reconstruct DNA fragments, i.e., contigs, from short reads by assembling overlapping reads. By comparing the assembled contigs to the reference genome, the genomic regions with discordant copy numbers are then identified (Figure 1d). This direct assembly of short reads without using a reference is called *de novo *assembly. Assembly can also use a reference genome as a guide to improve its computational efficiency and contig quality. AS-based methods have a minimum read coverage requirement to satisfy the algorithm and detect overlapping fragments, although high coverage will increase the complexity of short read assembly, especially for *de novo *AS-based tools.

The Cortex assembler was designed as a complete *de novo *CNV calling method [57]. It uses de Bruijn graphs to assemble overlapped reads from multiple sequencing samples into a single graph. The nodes and edges in the graph are marked in different colors to differentiate different samples. Then, it discovers CNVs by piping all the nodes from different samples together to find bifurcation structures, in which the sequence of the sample differs from the sequence of the reference genome. The branches to separate different colors represent SVs, e.g., insertions and deletions. Another *de novo *AS-based tool, Magnolya, that was published recently, also estimates copy numbers from two or more samples by utilizing a Poisson mixture model [58].

Without relying on read alignment, the AS methods potentially provide an unbiased approach to discover novel genetic variants ranging from the single base pair level to large structure variation. However, AS methods are rarely used in CNV detection in non-human eukaryotic genomes due to the low quality of the assembled contigs and their overwhelming demand on computational resources.

### The combinatorial approach

Although there has been great progress in each of the four categories of PEM, SR, RD, and AS approaches, and a growing number of tools have been developed, none of them have been able to detect the full spectrum of all types of CNVs with high sensitivity and specificity. With distinct advantages and weaknesses, these four strategies could be complementary to each other. PEM-based methods can detect all types of SVs (e.g., deletion, novel insertion, translocation, inversion, interspersed and tandem duplication) [59], especially for deletions of less than 1 Kb. However, PEM tools cannot accurately estimate the actual numbers of copies, and they are not applicable to insertions larger than the average insert size of the library. In contrast, RD-based methods can accurately predict copy numbers, have good performance to detect large CNVs, and are applicable to both WGS and WES data. However, they are not applicable for detection of precise breakpoints, copy neutral events like inversions or translocations, or small CNVs (e.g., <1 Kb). For the AS-based tools, their exclusive advantage lies in that they do not require a reference genome as input, and, importantly, they allow the discovery of novel mutation sequences. However, AS-based tools require extensive computation and perform poorly on repeat and duplication regions. For the SR-based tools, they could detect breakpoints at base pair resolution and performed well on deletion and small insertions. However, SR-based tools have low sensitivity in regions with low-complexity, as they rely on unique mapping information to the genome. To overcome the inherent limitations and take advantage of different types of methods, a number of tools have been developed attempting to integrate multiple approaches from different categories to increase the performance in detecting CNVs and reduce false positive discoveries.

Five tools in Table 4 are able to use PEM and RD information to identify CNVs in populations, including SVDetect [60], CNVer [61], Genome STRiP [62], GASVPro [63], and inGAP-sv [64]. As aforementioned, combinatorial methods can take advantage of the uniqueness of multiple tools and, thus, can reduce more false positives than any method that is purely built on PEM or RD (Figure 1e). For example, PEM methods can report accurate breakpoints, but their efficiency is low when detecting large CNV regions (e.g., insertions longer than the insert size) or counting exact copy numbers. On the other hand, RD methods are advantageous to detect large CNVs but cannot report exact breakpoints. A combination of the two would enable accurate detection of CNV regions with exact breakpoints and spanning various lengths, especially for longer insertions that are undetectable by PEM. In addition, the independent identification of CNV regions helps the tools to reduce false positives.

SVDetect is the first tool to introduce read depth information to improve PEM-based detection of large SVs. Combining the breakpoint predicted by the PEM approach and the log-ratio from the read depth between the matched case and control samples, the tool makes final judgements on gain and loss events. CNVer is another tool that combines PEM and read depth information for CNV identification [61]. Similar to SVDetect, CNVer makes use of discordant read pair mapping information to identify breakpoints and read depth signals to identify aberrant genomic fragments. Notably, an additional advantage of CNVer is that it employed an ambiguous mapping strategy to allow better use of the reads that are mapped to multiple genomic locations. In this way, higher sensitivities in repeat and duplication regions can be achieved by CNVer. Genome STRiP is used to identify SVs/CNVs in large populations such as the date generated in The 1000 Genome Project [62]. To reduce false positives in deletion identification, the tool requires that the deletion identified by the PEM approach should also be confirmed by the lower read depth in the same genomic regions.

The GASV-Pro is another successful example of a combinatorial method. It was upgraded from a single PEM-based tool, GASV (Geometric Analysis of Structural Variants), and incorporated read depth information to combinatorially call CNVs. GASV proposed a novel geometric approach to cluster the discordant mappings from paired reads [65]. In this approach, the breakpoint region is represented as a polygon in a plane. This tool can further identify the accurate boundary supporting the same variant using an efficient plane sweep algorithm to detect the intersections of the polygons. The advantage of GASV is that it can integrate both arrayCGH and paired-end sequencing reads to call SVs, including CNVs. Different from GASV, GASVPro implements a probabilistic model to integrate both read pairs and read depth information [63]. Using a Markov Chain Monte Carlo (MCMC) approach, GASVPro can map all the reads to all possible locations. The breakend read depth (BeRD) information is defined as the dropped read depth at the breakpoints. Incorporating the BeRD signals, GASVPro uses the amount of discordant read pairs to determine the probability of each detected CNV. Another combinatorial tool, inGAP-SV, can not only detect CNVs by combining read pairs and read depth information but also provide a genome browser-like visualization solution for large and complex SVs [64]. Similar to the beRD concept in GASVPro, inGAP-SV first uses the drops in read depth as signals to defined SV "hotspots." Then, a heuristic search approach is applied on these hotspots to classify SVs using the PEM approach. In summary, there are at least two key features for inGAP-SV: capacity to identify different types of CNVs and customized visualization.

NovelSeq is the first tool to combine PEM and SR approaches and was developed specifically to detect novel insertions in the genome [66]. It starts with the unmapped reads and assembles them to form contigs. A clustering algorithm is then applied to the assembled contigs to identify potential insertion events supported by these unmapped reads. HYDRA is a tool that combines PEM and local *de novo *assembly to detect SVs and was originally applied in the mouse genome. It also relies on discordant mate pairs for SV detection, using both short and long reads as input [67]. HYDRA applies a soft clustering approach to identify putative SVs and breakpoints and therefore has the potential to be very useful to identify SVs in repeat and duplication regions in the human genome.

## Challenges and perspectives

Intensive efforts in recent years to develop computational tools for CNV detection have resulted in 48 tools, as described in this paper, which target specific ranges of CNVs by utilizing different computational strategies. Systematic comparison showed that only small overlapping SVs/CNVs are identified based on different tools in population-scale genome sequencing [59]. These small overlaps imply there may be different scales of SVs/CNVs in the human genome. Recently, distinct SV patterns were observed in experiments that used two libraries with different insert sizes [68]. This observation indicates the importance of choosing appropriate tools for a specific experimental design.

Despite improvements to NGS technologies and CNV-detecting tools, the identification of low coverage CNVs still remains a challenge. Although the RD-based approach has to correct distortions caused by NGS biases, the relationship between the read count and true copy number can be distorted by several effects. The PCR process is known to be one major cause of this distortion, where genomic fragments with a lower PCR amplification rate often result in less reads [69,70]. Furthermore, sequencing process can also introduce system noise [55,71]. It was reported that NGS has lower sequencing coverage in regulatory regions [71]. In particular, the capability of exome capture in the library preparation process complicates the connection between true copy number and read count for WES data. All these factors contribute to the difficulty in detecting the low coverage copy number. For the regions with a low read count, it is hard to articulate if it is indeed due to the low copy number or if it is a consequence of the aforementioned processes. In the following section, we focus on the RD-based strategy to discuss potential noise from NGS data and how to overcome these shortcomings using specific normalization. Since most of the PEM- and SR-based tools described here are general tools to detect SVs, their limitations and strengths were extensively reviewed previously [16].

One well-investigated bias in RD-based methods is related to GC content, the percentage of guanine and cytosine bases in a genomic region. GC content varies markedly along the human genome and across species and has been found to influence read coverage on most sequencing platforms. In a recent study of the Illumina HiSeq and Genome Analyzer systems [72], for example, a positive correlation between read coverage and GC content was observed when GC percentage is within the spectrum of 24 to 47%. To correct the GC bias, a widely used strategy in existing RD-based tools is to partition the genome into windows/bins of either fixed or various sizes [28,31,41]. The number of reads mapped to each window are counted and then normalized based on the GC content of this windowed region. The adjusted read count is then used as an indicator of the copy number of the corresponding genomic region. In NGS applications that involve a pair of disease and normal tissues taken from the same patient and sequenced on the same platform, the effect of GC bias can be effectively cancelled out by comparing the paired genomes directly at each region [31,33,41], thereby taking advantage of the similarity of GC bias between the two paired samples. For instance, CNAseg implemented an algorithm locally weighted scatterplot smoothing (LOWESS) regression to adjust the GC bias for matched samples in each 10-Kb window. Moreover, Discrete Wavelet Transform (DWT) is used to smooth the signals of mapped reads, which helps to obtain more accurate estimation of relative copy numbers between case and control samples.

Another major bias that affects CNV calling when using the RD-based approach originates from read alignment. In the alignment step, a significant portion of reads are mapped to multiple positions due to a short read length and the presence of repetitive regions in the reference genome. A variety of strategies have been proposed to assign ambiguously mapped reads to the reference genome, and each suggested approach has its own pros and cons, affecting read coverage and, hence, copy number in a different way. Some methods, e.g., BIC-seq [41] and SegSeq [33], completely ignore ambiguously mapped reads. Apparently, by counting only the uniquely mapped reads, these methods are not applicable to detect CNVs in homologous genomic regions. Other methods, e.g., CNVnator [40], randomly assign an ambiguous read to one of all possible positions to which this read is mapped by the aligner. Though capable of identifying copy numbers in repetitive genomic regions, this strategy suffers from false positive detection as a consequence of the random placement of ambiguously mapped reads. One possible direction to improve mapping sensitivity is to employ a soft clustering approach, which allows multiple good mappings [54]. Despite these improvements on mapping algorithms, all of the methods described here are still problematic in repeat regions. Allowing mismatches in repeat regions may increase sensitivity in the future.

To assist researchers in selecting the proper NGS tool(s) for their CNV studies, here, we summarize our discussion of SV-detecting tools collected in this paper. Several tools, such as JointSLM and ExoCNVTest, are applicable to detect common CNVs shared among multiple samples, while CoNIFER and XHMM are good for rare CNV identification from a population of WES samples. Additionally, Pindel is an effective tool for the detection of small deletions of lengths less than 300 bp, while RD-based tools are more suitable to characterize large CNV events. As for CNV breakpoints, SVseq is designed to identify deletion with exact breakpoint information from low coverage NGS data [73]. NovelSeq is specific to locate novel insertion. The tool inGAP-SV facilitates researchers to visualize the detected interesting CNV regions.

Compared with CNV detection using only single tool, the combination of different software has proved effective in improving CNV prediction accuracy. For example, a study that combined the results of BreakDancer and VariationHunter demonstrated higher sensitivity and specificity to detect SVs/CNVs [17]. This result implies that the combination of different PEM tools can help detect high confidence CNVs. In Table 4, we show that PEM-based tools are often combined with RD and AS approaches to improve both sensitivity and specificity for CNV detection using WGS data. Unlike WGS data, the discontinuous reads from WES limit the application of PEM-based methods. For example, the insert size for pair-end reads in WES may not be long enough to detect CNV using PEM-based methods. However, the SR-based tool SLOPE has good performance to pinpoint translocation events by focusing on small, targeted genomic regions. By recognizing the successful application of a combinatorial approach to detect CNVs from WGS data, the similar strategy to combine RD and SR features may provide more reliable CNV predictions from WES data [74]. In summary, incorporating various signatures to support the same CNV can dramatically improve both the statistical power and resolution of CNV size and breakpoints compared to each different strategy individually.

Competing for higher sequencing throughput, data accuracy and longer read lengths, today's sequencing technologies will continue to advance rapidly. One prominent development is the creating of third generation sequencing (TGS) technologies, which promise to provide dramatically longer read lengths. The first commercial TGS sequencer launched by PacBio (PacBio RS single molecule sequencing) can now produce reads of an average length 1300 bp, significantly longer than that of any NGS platforms today. Longer reads will greatly ease read alignment and CNV detection in repetitive regions of genome [75,76]. These longer reads will significantly reduce mapping errors due to incorrect sequencing. The increased size of the short read will also strengthen the statistical power of the RD methods. In addition, all these improvements on mapping quality will benefit PEM methods to avoid false positives caused by chimera in the genome. For the SR methods, these longer reads may also ameliorate their inherent limitation of only detecting insertion events shorter than the average read length. Additionally, longer reads may improve assembly quality when implementing AS methods. The development of high resolution CNV detection tools is an iterative process of integrating new technologies' features and novel computational algorithms. Though these processes require committed efforts from industry-academy collaborative work, the fruitful understanding of human CNVs in healthy and disease conditions will be a worthy endeavor, which may not only allow discrimination of driver mutations for pathogenesis of Mendelian and complex diseases but also help to develop personalized medicine in the future.

## Abbreviations

AS: *de novo *assembly; BAM: Binary version of a SAM file; CB: Combinational approach; CBSm Circular binary segmentation; CGH: Comparative genome hybridization; CNV: Copy number variation; EM: Expectation maximization; FASTQ: Nucleotide sequence with its corresponding quality scores in text format; FISH: Fluorescence *in situ *hybridization; HMM: Hidden Markov model; INDEL: Insertion and deletion; MSB: Mean shift-based method; NGS: Next-generation sequencing; PEM: Paired-end mapping; R: R language for statistical analysis; RD: Read depth; SAM: Sequence Alignment/Map file format; SLM: Shifting level model; SNP: Single-nucleotide polymorphism; SNV: Single-nucleotide variant; SR: Split read; SV: Structural variant; WES: Whole-exome sequencing; WGS: Whole-genome sequencing.

## Competing interests

The authors declare that they have no competing interests.

## Authors' contributions

MZ, PJ and ZZ conceived of the study. MZ primarily wrote the manuscript. QW1, QW2, PJ and ZZ participated in manuscript writing. All authors read and approved the final manuscript.

## Declarations

The publication costs for this article were funded by the corresponding author.

This article has been published as part of BMC Bioinformatics Volume 14 Supplement 11, 2013: Selected articles from The Second Workshop on Data Mining of Next-Generation Sequencing in conjunction with the 2012 IEEE International Conference on Bioinformatics and Biomedicine. The full contents of the supplement are available online at http://www.biomedcentral.com/bmcbioinformatics/supplements/14/S11.
